# A National Assessment of Cancer Center Shared Resources Metrics: Results from the First NACCSR Survey

**DOI:** 10.7171/001c.166574

**Published:** 2026-09-01

**Authors:** Jerry Edward Chipuk, Fei Ye, Jai N. Patel, Steven J. Madore, Crystal F. Kline, Matthew Nowacki, Andrés Chousal Cantú, David S. Goerlitz, Kevin K.C. Lloyd, Jay W. Fox, George S. Grills

**Affiliations:** 1 Tisch Cancer Center Icahn School of Medicine at Mount Sinai; 2 Deptartment of Oncological Sciences Icahn School of Medicine at Mount Sinai; 3 Sylvester Comprehensive Cancer Center University of Miami Miller School of Medicine; 4 Department of Public Health Sciences University of Miami Miller School of Medicine; 5 Wake Forest Baptist Comprehensive Cancer Center; 6 Wake Forest University School of Medicine; 7 University of Florida Health Cancer Institute; 8 The Ohio State University Comprehensive Cancer Center; 9 Department of Oncological Sciences Icahn School of Medicine at Mount Sinai; 10 Lombardi Comprehensive Cancer Center Georgetown University Medical Center; 11 UC Davis Comprehensive Cancer Center University of California, Davis; 12 University of Virginia Comprehensive Cancer Center University of Virginia Health System

**Keywords:** National Alliance of Cancer Center Shared Resources, cancer center, shared resources, core facilities, management, NACCSR, NCI designation, survey

## Abstract

Tracking core metrics is a critical component of centralized core facility management and is essential for ensuring that individual cores are efficiently operated, effectively evaluated, and optimally catalyze research. The National Alliance of Cancer Center Shared Resources (NACCSR) is a consortium of shared resource leaders from the National Institutes of Health (NIH) National Cancer Institute (NCI) designated Cancer Centers. The NACCSR Core Metrics Committee (CMC) includes shared resource leaders from a dozen NCI-designated cancer centers. Presented here are the results of a recent CMC survey aimed at identifying core metrics critical to cancer center shared resources, including operations, usage, financials, and scientific impact. The end goals of this survey are to benchmark cancer center core metrics and to provide a base for recommendations on best practices for optimizing the tracking, evaluation, and presentation of core metrics. Moreover, these survey findings will inform evidence-based recommendations to the NCI on the essential core metrics to be incorporated into both the individual shared resource sections and the centralized shared resource management components of NCI P30 Cancer Center Support Grants.

## Introduction

Shared resources (SRs) at National Cancer Institute (NCI)-designated cancer centers catalyze and promote cancer research by providing ready access to cost-effective, state-of-the-art research technologies and services, as well as high-level scientific and technical expertise. NCI-designated cancer center SRs (also called cores or core facilities) support innovative transdisciplinary research and facilitate novel discoveries in basic, translational, and clinical research, leading to new approaches to the prevention, diagnosis, and treatment of cancer.

Formed in 2025, the National Alliance of Cancer Center Shared Resources (NACCSR) is a consortium of SR leaders from NCI-designated cancer centers and designation-seeking cancer centers across the United States. NACCSR members work collaboratively to advance SR-supported cancer research. The NACCSR promotes cross-center SR strategic partnerships and collaborations to accelerate discovery and translation; to facilitate the exchange of best practices, data, and emerging technologies; and to enhance access to advanced services, instruments, and training. The efforts of NACCSR members drive innovation and optimization in SR management, business sustainability, and research impact. The NACCSR serves as a unified voice to champion the essential role of SRs in cancer research.

The NACCSR Core Metrics Committee (CMC) was established in 2025, composed of SR leaders from 12 NCI-designated cancer centers. The CMC conducted a cross-sectional, center-level survey of SR management in 2025, with the objective of providing a better understanding of how cancer center SRs are managed and how and what SR metrics are tracked. The survey aimed at identifying core metrics that are critical to cancer center SRs, including information on oversight structure, operations, usage, financials, and scientific impact. The end goals of this survey were to benchmark cancer center core metrics and to provide a base for recommendations on best practices for optimizing the tracking, evaluation, and presentation of SR metrics.

Prior surveys of life sciences or biomedical SRs covered all types of academic institutions,^1–3^ highlighted specific types of institutions,^4–6^ or focused on types of SRs based on services provided,^7–9^ but did not annotate which SRs are at cancer centers. Furthermore, prior studies on defining, tracking, reporting, and optimizing SR metrics^10–13^ covered all types of core facilities without focusing on best practices specifically for cancer center SRs. To our knowledge, this is the first published study on core metrics and management that focuses on cancer center SRs.

## Methods

### Survey Data Collection

The NACCSR CMC developed and posted an online core metrics survey in May 2025 using Qualtrics. An invitation to participate in this survey, along with a link to the survey, was emailed to the leaders of SR management (e.g., Associate Directors for SRs) at 85 cancer centers, including all 73 NCI-designated cancer centers, plus 12 other cancer centers that are actively seeking NCI designation. Responses were collected over four weeks (05/19/25–06/20/25).

### Survey Data Analysis

Analyses of survey responses were unweighted and performed on one response per cancer center. Continuous and count-type variables were summarized with medians and interquartile ranges; categorical variables with counts and percentages. Response denominators vary across items due to partial nonresponse, so percentages are reported based on the number of centers that answered each question. A nonparametric Wilcoxon rank-sum test was used for comparisons involving continuous or count outcomes and binary grouping factors (e.g., type of advisory committee: individual, umbrella, both, or neither; or organizational structure: consortium, freestanding, matrix, or other). Associations between categorical variables (e.g., NCI designation vs. policy indicators) were evaluated using chi-square (χ^2^) test or Fisher’s exact test as appropriate. Odds ratios are reported with 95% confidence intervals (CIs) when appropriate. For continuous outcomes (e.g., percent SR cost recovery and Shared Resources Management [SRM] staffing count), we fit linear regression models with prespecified covariates, including number of Cancer Center Support Grant (CCSG)-supported SRs, years since first NCI designation, and NCI designation category. For binary outcomes (e.g., presence of an SR management team, access-prioritization policy), we used logistic regression (binomial link). We fit prespecified univariable models for each candidate predictor and parsimonious multivariable models (e.g., NCI designation plus number of CCSG-supported SR) to avoid overfitting given sample size and sparsity. Models exhibiting quasi-complete separation were treated as descriptive. Satisfaction with institutional support (5-point ordinal scale) was analyzed using proportional-odds ordinal logistic regression. Univariable models were fit for each candidate predictor. The multivariable ordinal model was limited to two degrees of freedom (e.g., percentage of SR budget covered by CCSG support and number of SR management personnel) due to the limited effective sample size. Given outcome missingness, analyses were restricted to complete cases (no imputation; denominators are reported per analysis) to avoid model-based extrapolation under unverifiable missing at random (MAR) data. Two-sided tests used a significance level of 0.05 without multiplicity adjustment, consistent with the survey’s benchmarking purpose and the exploratory nature of the analysis. All statistical analyses were conducted in R version 4.5.0 and documented in an R Markdown HTML report for reproducibility.

## Results

### Respondent Demographics

Understanding the demographic of the responders to the NACCSR survey is essential to benchmarking SR data results, identifying trends, and informing future initiatives to improve SRs and their management. The survey was distributed to 85 cancer centers, including all NCI-designated cancer centers (73) plus additional cancer centers (12) that are actively seeking NCI designation. Survey responses were received from 57 cancer centers (i.e., 67% response rate), providing a cross-sectional benchmark of SR governance, evaluation, and financial sustainability across NCI-designated and designation-seeking institutions. The 57 responders included NCI-designated comprehensive cancer centers (72%), NCI-designated cancer centers (20%), and cancer centers seeking NCI designation (8%) (Figure 1A). The organizational structure of the responding centers included matrix (79%), freestanding (11%), and consortium cancer centers (10%) (Figure 1B). The median number of years since NCI designation was approximately 35 years (Figure 1C). The median number of SRs per cancer center was eight, with 66% of centers supporting six to 10 SRs (Figure 1D). In a cross-sectional depiction of NCI-designated centers, the number of CCSG-supported SRs showed a modest, nonlinear association with years since first designation. The locally estimated scatterplot smoothing (LOESS) fit (a nonparametric method for smoothing a series of data in which no assumptions are made about the underlying structure of the data) rises from early tenure to approximately 20–30 years, plateaus through mid-tenure, and increases again beyond ~45 years. Substantial dispersion at all tenures indicates that longevity explains only a limited proportion of between-center variability, and may reflect confounding center size, CCSG budget, programmatic scope, or organizational structure (Figure 1E).

**Figure:**
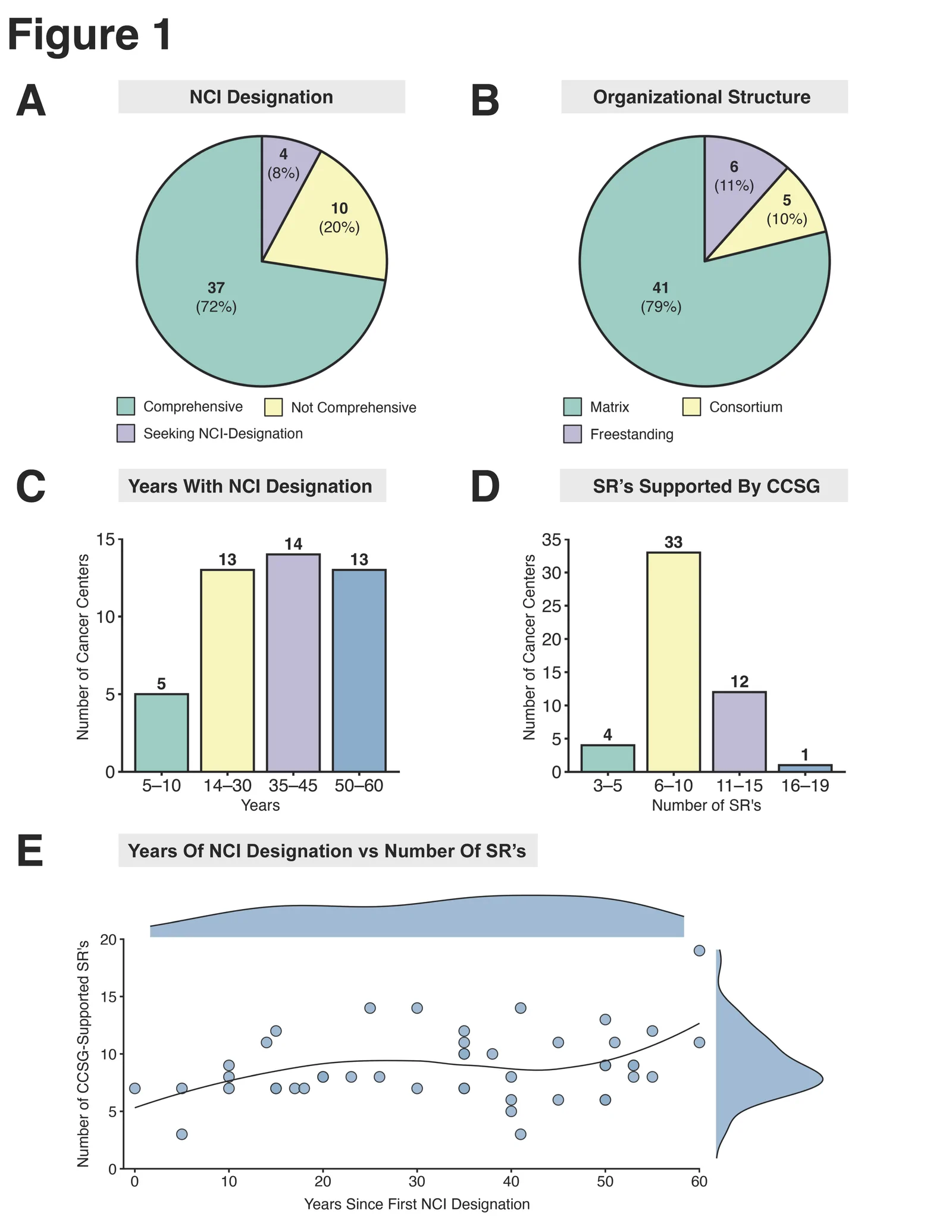
Figure 1. **Demographic Composition of the NACCSR Survey 2025 Responders.** (A) NCI designation of the respondent’s cancer center (n = 51 responses). (B) Organizational structure of the respondent’s cancer center (n = 51 responses). (C) Years that the respondent’s cancer center has NCI designation (n = 45 responses). (D) Number of SRs supported by the CCSG at the respondent’s cancer center (n = 50 responses). (E) Relationships between years since NCI designation and the number of CCSG-supported SRs. Each point represents one cancer center, plotting years since the first NCI designation (x-axis) versus number of CCSG-supported SRs (y-axis). The solid black curve is a LOESS smooth summarizing the trend. Shaded marginal densities along the top and right display the univariate distributions of the x- and y-variables, respectively. Points are shown in neutral gray with equal weight.

### Shared Resources Management Structure

**Figure 2.**
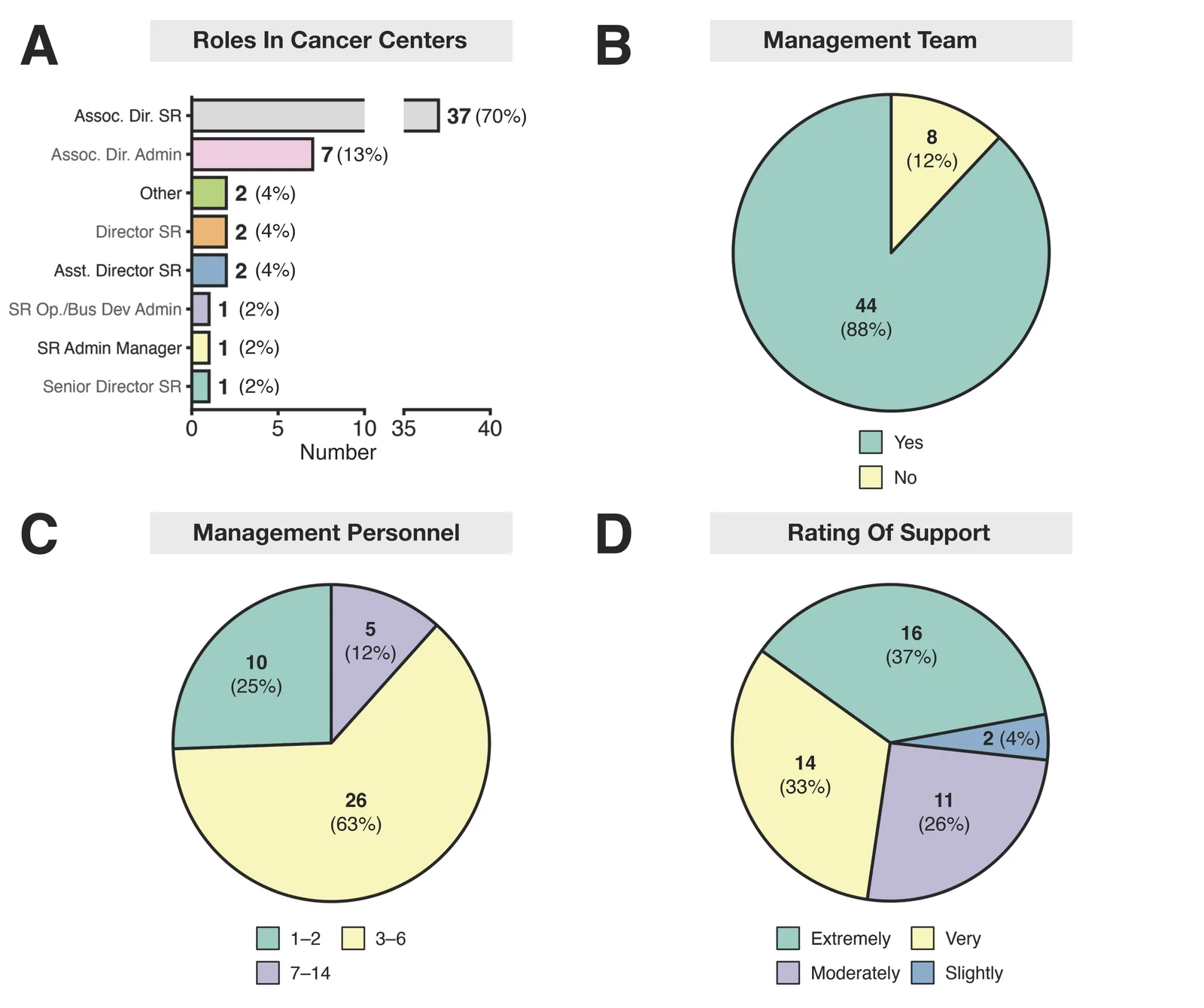
**SRM Structures within the NACCSR Survey 2025 Responders’ Cancer Centers.** (A) Respondent’s role in their cancer center (n = 53 responses). (B) Establishment of an SRM team at the respondent’s cancer center (n = 50 responses). (C) Number of SRM team personnel at the respondent’s cancer center (n = 43 responses). (D) Respondent’s rating of cancer center support for SRM (n = 43 responses).

A key component of ensuring SRs are effective in supporting cancer center research programs is the SR management structure. A majority of survey responders hold the title of Associate Director for SRs (70%) or Associate Director for Administration (13%) (Figure 2A). Within the cohort of responders, 88% indicated the existence of a formal centralized SRM team at their cancer center (Figure 2B). A total of 63% of respondents reported three to six personnel supporting SRM operations, 25% reported one to two personnel, and 12% reported seven to 14 personnel (Figure 2C). The survey asked participants to assess their satisfaction with their respective cancer center’s support for SRM. A total of 37% of the respondents rated their cancer center’s support as extremely supportive, 32% as very supportive, 26% as moderately supportive, and 5% as slightly supportive (Figure 2D). In a multivariable ordinal logistic regression model, the level of CCSG-supported SR effort was significantly associated with higher satisfaction ratings for institutional support of SRM, adjusted for number of SRM personnel. For every 1% increase in CCSG-supported effort, the odds of receiving a higher satisfaction rating increased by approximately 12% (Odds Ratio [OR] = 1.12; 95% CI: 1.02–1.23; *p* = .024). A higher number of SRM personnel showed a tendency toward a positive association with more positive ratings of center support in the same model (OR = 1.61; 95% CI: 0.92–2.80), though not reaching statistical significance (*p* = .093).

### Measuring SR Impact and User Satisfaction

Survey responses were used to ascertain common practices to measure the impact of CCSG-supported SRs along with SR user satisfaction. The top three metrics used to determine an SR’s impact on cancer research were: (1) the number of cancer-relevant publications facilitated by the SR, (2) the number of cancer-relevant new grant awards supported by the SR, and (3) the novel capabilities of the SR (Figure 3A). The most frequent means to gather cancer member feedback on SRs were user surveys (94%), cancer center leadership meetings (82%), and an SR advisory committee (80%) (Figure 3B). A broad array of methods are used to identify publications supported by SRs (a CCSG reporting requirement), including use of National Institutes of Health/National Library of Medicine (NIH/NLM) PubMed to search for acknowledgment of SRs (including by citing an SR’s Research Resource ID) in cancer-relevant publications by cancer center members, SR personnel as authors in a publication, surveys asking SR users which of their publications were facilitated by specific SRs, and manual tracking of publications by personnel of individual SRs (Figure 3C). The top metrics commonly tracked regarding utilization and impact of an individual SR included the total number of users, the number of cancer center member users, publications facilitated, and the amount of SR revenue. Additional metrics included educational impact, grant dollars supported, new grant awards facilitated, and multi-SR support for research (Figure 3D). The metrics most often used to determine whether an SR qualifies for CCSG support were consistent across respondents, with 92% utilizing the SR’s impact on cancer-relevant research, 84% measuring the percentage of cancer center members using the SR, and 82% determining the number of cancer center members using the SR (Figure 3E).

**Figure:**
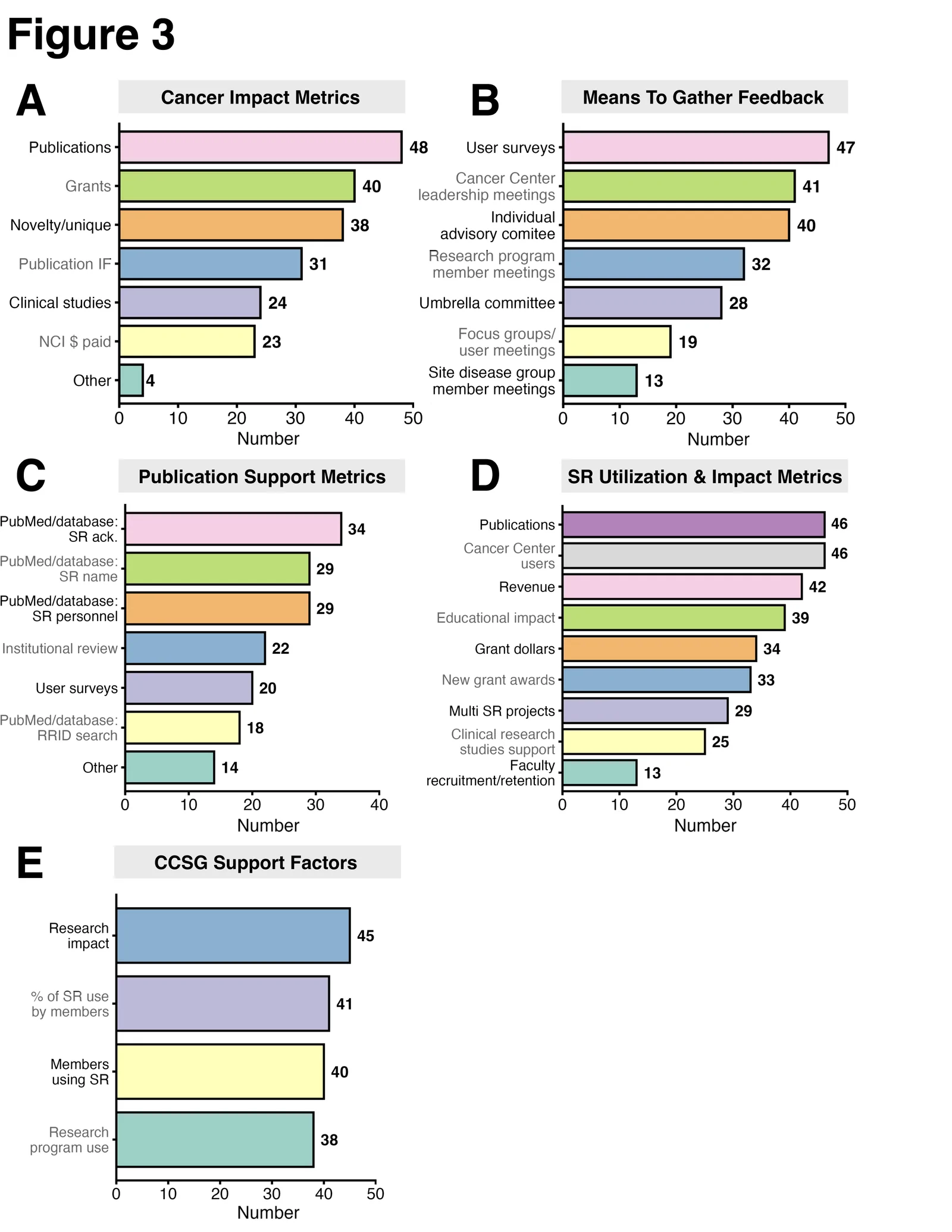
Figure 3. **Determinations for How SR Impact Metrics are Measured.** (A) Metrics used to determine an SR’s impact on cancer research (n = 49 responses). (B) Mechanisms to gather cancer member feedback on SRs (n = 50 responses). (C) Methods to determine the number of publications supported by a SR (n = 47 responses). (D) Metrics tracked for utilization and impact of each SR (n = 49 responses). (E) Metrics used to determine whether a SR qualifies for CCSG support (n = 49 responses).

**Figure 4.**
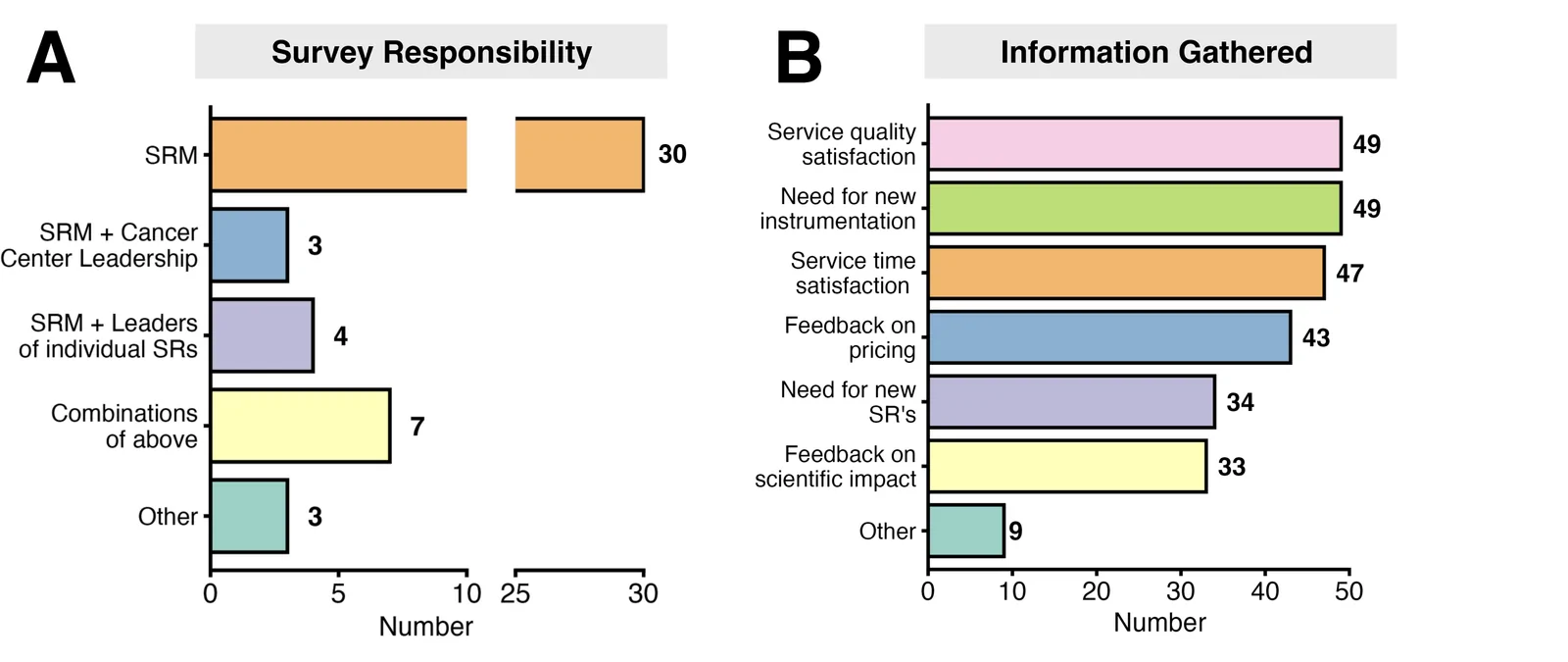
**Common Practices for SR User Surveys.** (A) Individual(s) responsible for sending SR user surveys at the respondent’s cancer center (n = 47 responses). (B) Information gathered by SR user surveys (n = 49 responses).

SRM was often responsible for taking the lead in sending SR user surveys (>64% of respondents indicated SR user surveys were either primarily or exclusively the responsibility of the SRM team), sometimes in combination with leaders of individual SRs, cancer center leadership, or other research administration (e.g., medical school) leadership (Figure 4A). The types of information commonly collected by SR user surveys include the need for new services and instrumentation, satisfaction with service quality, satisfaction with service turnaround time, and feedback on pricing (Figure 4B).

### SR Advisory Committee Structure

**Figure 5.**
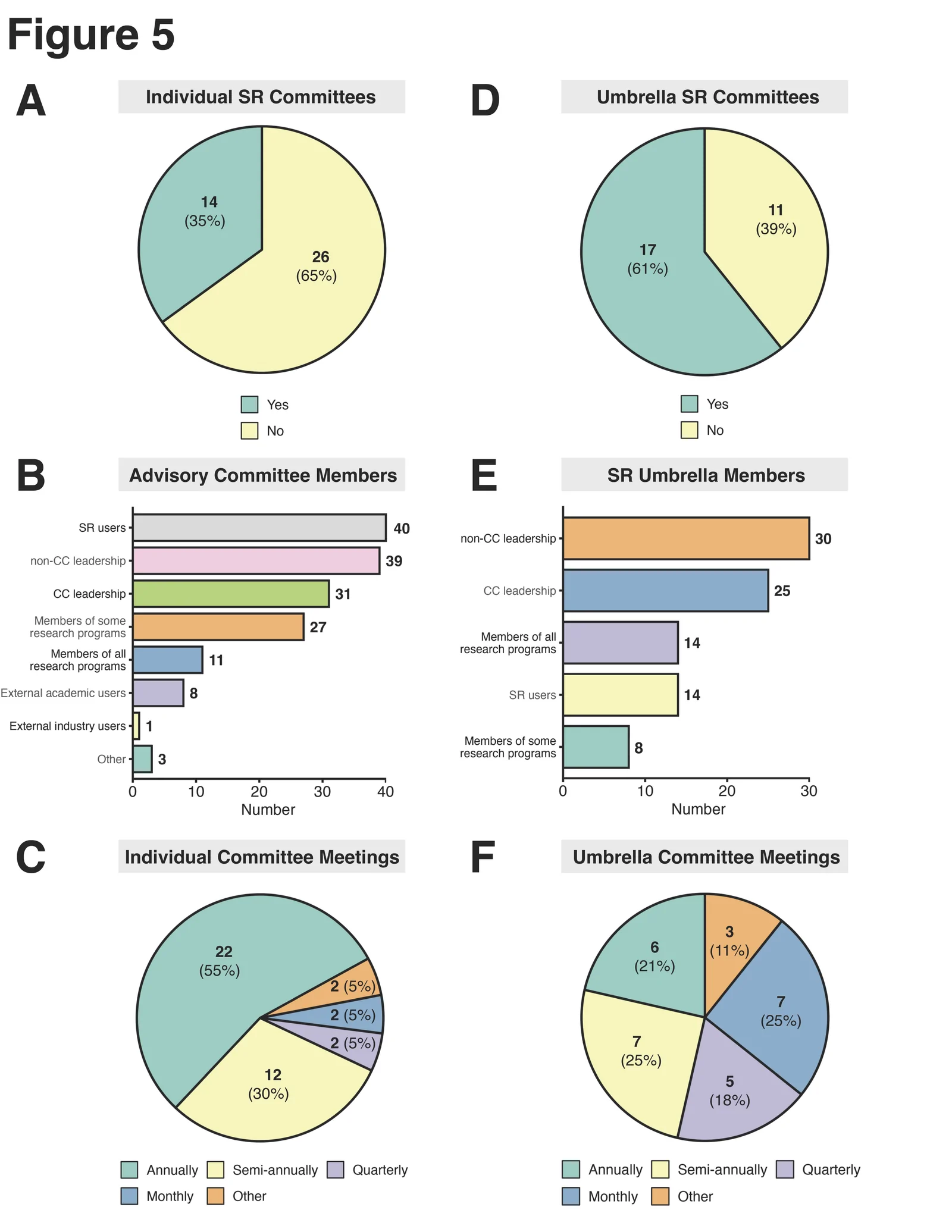
**Structure and Operations for SR Advisory Committees.** (A) Establishment of individual advisory committees dedicated to each CCSG-supported SR (n = 40 responses). (B) Composition of the individual advisory committees dedicated to each CCSG-supported SR (n = 40 responses). (C) Frequency of meetings for the individual advisory committees dedicated to each CCSG-supported SR (n = 40 responses). (D) Establishment of an umbrella advisory committee dedicated to all CCSG-supported SRs (n = 28 responses). (E) Composition of the umbrella advisory committee dedicated to all CCSG-supported SRs (n = 28 responses). (F) Frequency of meetings for the umbrella advisory committee dedicated to all CCSG-supported SRs (n = 28 responses). CC, cancer center.

The survey included questions on the existence, composition, and frequency of meetings of SR advisory committees, including individual advisory committees dedicated to each CCSG-supported SR and “umbrella” advisory committees dedicated to all CCSG-supported SRs at the institution. A total of 35% of the respondents indicated that they have individual advisory committees for each of their individual CCSG-supported SRs (Figure 5A), with these committees often comprised of cancer center leadership, non-cancer center leadership, SR users, and a representative from at least one CCSG-supported research program at the cancer center (Figure 5B). These advisory committees usually meet annually (55%) or semi-annually (30%) (Figure 5C). The majority of cancer centers (61%) have an umbrella advisory committee dedicated to all the CCSG-supported SRs at the institution (Figure 5D). The membership of these umbrella committees typically include cancer center leadership, non-cancer center leadership users, users, and representatives from all the CCSG-supported research programs at the institution (Figure 5E). The frequency of meetings of umbrella advisory committees varied, including annually (21%), semi-annually (25%), quarterly (18%), and monthly (25%) (Figure 5F). In exploratory comparisons, the number of CCSG-supported SRs did not differ meaningfully between centers that reported having individual SR-specific advisory committees and those that did not (median 8 vs. 8; mean 8.70 vs. 7.22; Wilcoxon *p* = 0.206). This suggests that adoption of individual advisory committees is not simply a function of SR portfolio size but likely reflects local management preferences or institutional practices.

### SR Financial Management and Business Sustainability

**Figure 6.**
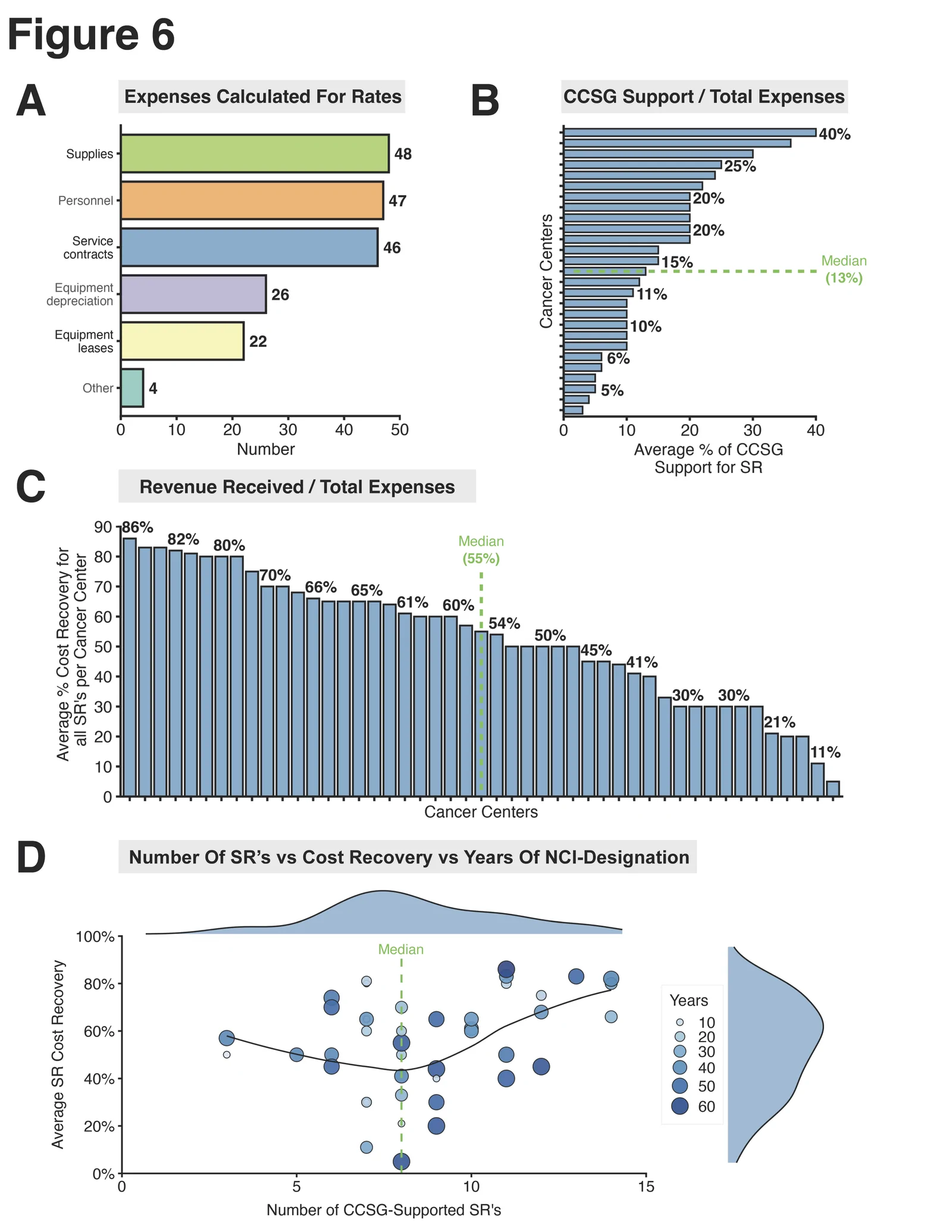
**Financial Relationships Related to SR Operations.** (A) Expenses included in the calculation of SR rates (n = 49 responses). (B) Average percentage of CCSG support for SRs, defined as CCSG support divided by total expenses (n = 27 responses). (C) Each bar is the average percent cost recovery for all the SRs at a cancer center, defined as the total of SR revenue received divided by the total of SR expenses at that cancer center (n = 47 responses). (D) Relationships between the number of CCSG-supported SRs and the average SR cost recovery. Each point is one cancer center; the x-axis shows the number of CCSG-supported SRs, and the y-axis shows the center’s average SR percent (%) cost recovery. Point size encodes years since initial NCI designation. The solid line is a LOESS smooth summarizing the trend; the vertical dashed line marks the median number of CCSG-supported SRs. Gray marginal density plots show the distributions of the x- and y-variables.

To better understand the financial management and business sustainability of SRs, the survey collected data on what expenses are used to determine SR fee-for-service rates, CCSG contributions to SR budgets (e.g., CCSG support used to offset the cost of SR services to cancer center members), and the percent cost recovery of all SRs combined within a cancer center. Expenses most often considered when determining SR user rates include supplies (98%), personnel costs (96%), and service contracts (94%). Other expenses were less commonly included in SR user rates, such as equipment depreciation (53%) and equipment leases (45%) (Figure 6A). The average percent of CCSG support for all SRs combined in a cancer center varied from 3% to 40%, with a median of 13% support (Figure 6B).

The average percent cost recovery for all SRs combined in a cancer center ranged from 5% to 86%, with a mean of 53% cost recovery and a median of 55% cost recovery (Figure 6C). In a univariable analysis, each additional CCSG-supported SR in a cancer center is associated with a 2.6% increase in the average percent cost recovery of all the SR combined (95% CI: 0.46–4.80; *p* = .019). When adjusted for years of NCI designation in a multivariable linear regression, the number of CCSG-supported SRs remained significantly associated with higher cost recovery (*p* = .031). Adjusting for years of NCI designation, cancer centers with >8 (median) CCSG-supported SRs have 22% higher SR cost recovery than those with 8 or fewer CCSG-supported SRs (95% CI: 1.8–42.5; *p* = .034) (Figure 6D). The LOESS curve also suggests a nonlinear pattern, with cost recovery dipping near the median of 8 SRs and increasing above the median. Years of NCI designation were not significantly associated with cost recovery (*p* = .29) (Figure 6D).

### SR Data Management and SR Access

#### Data management

The survey asked who covers the cost of data generated by SRs. Respondents from 24 cancer centers replied to this question. A total of 42% (10 respondents) indicated that storage of data generated by SRs is paid for by their institution, 46% (11 respondents) indicated that data storage is paid for by a combination of the SR users and the institution, and 12% (three respondents) indicated that their SR users are solely responsible for the cost of data storage at their institution. The majority indicated that their SRs have guidelines on how long certain data types are stored. A total of 79% of respondents indicated that their cancer center has comprehensive policies for managing data generated by their SRs.

#### SR priority access policies

A total of 83% of respondents from 48 cancer centers confirmed that their institution has policies for prioritization of access to SR services for cancer center members. A total of 17% of these respondents indicated that their institution does not have a formal SR priority access policy.

## Discussion

The NACCSR Core Metrics Survey provides a comprehensive overview of the current landscape of SRM, SR evaluation, and SR business sustainability across NCI-designated cancer centers and designation-seeking cancer centers. By leveraging the NACCSR collaborative network, we have identified prevailing trends, strengths, and potential areas for improvement in the stewardship of SRs that are critical to advancing cancer research nationwide.

This survey offers a snapshot of SR management practices that are broadly consistent in their goals but heterogeneous in implementation. Most centers report having a formal SR management infrastructure and routinely evaluate SR impact using a common set of utilization and cancer-relevance indicators, typically complemented by regular user-satisfaction feedback. At the same time, organizational structure and oversight models show substantial center-to-center variation, particularly advisory committee structures and approaches to tracking SR-supported publications. Financial practices likewise display wide differences in the balance between institutional support and user fee-based cost recovery.

A broad array of methods are used to identify publications supported by SRs (Figure 3C), including using PubMed searches and using member surveys to determine SR authorship and acknowledgment. Of note, only a third of the respondents leverage assignment and attribution of SR’s Research Resource IDs to track publications supported by their SRs. This is a potential area to define and promote best practices. Research Resource Identifiers are part of a national initiative^14,15^ to help researchers cite key resources and thus facilitate research rigor and reproducibility. This initiative is supported by major journals and funding agencies.

The top metrics commonly tracked to evaluate the use and impact of SRs (Figure 3D) include the total number of users, the number of cancer center member users, the publications facilitated, and the revenue received. Fewer centers determine an SR’s impact on cancer research by using metrics such as existing grants supported, new grant awards facilitated, clinical research studies supported, coordinated multi-SR support for research, educational impact, or the SR’s impact on faculty recruitment and retention. This is another area to potentially define and promote best practices.

Exploratory modeling suggests that greater CCSG investment and a larger CCSG-supported SR portfolio are each associated with more favorable business sustainability indicators, independent of the number of years of NCI designation. One plausible interpretation of this link is an economy-of-scale effect: centers supporting more SRs likely have larger or more diverse user bases, higher aggregate level of use, and more centralized SRM infrastructure, all of which can improve rate setting, billing efficiency, and utilization tracking, translating into higher overall cost recovery. The number of CCSG-supported SRs remained significantly associated with higher cost recovery after adjusting for years of NCI designation, suggesting that this is not simply a maturity or “older centers recover more” story. Instead, the number of CCSG-supported SRs may be acting as a proxy for operational scale and complexity that requires more formalized financial management. Figure 6E shows a dip near the median and higher recovery above it, suggesting a possible threshold where centers transition from maintaining a core set of siloed SRs to running a larger, more integrated SR ecosystem with better cost accounting and recovery leverage. At the same time, these results should be viewed as hypothesis-generating given the moderate sample size and unmeasured confounding factors (e.g., center size, local cost structure, SR mix, and institutional pricing policies), and this survey could not reliably model nonlinearity beyond a median split. The percent cost recovery for any SR is related to the types of services offered by the SR, the developmental stage of the SR and the SR’s services, and the amount of institutional support available to enable an SR to be readily accessible to members. Future surveys that stratify cost recovery by SR type and by other contextual factors will help clarify whether the observed relationship reflects true scale advantages or differences in SR composition and institutional expectations across centers.

The responses to open-ended questions in the survey highlighted several cross-cutting themes. Respondents emphasized the value of centralized SRM with the flexibility to scale, add, or discontinue SRs as cancer center member needs change. Many also pointed out the increasing equipment and operational costs of SRs and advocated for stronger cross-center SR collaboration to reduce redundancy and increase cost efficiencies. This could include the establishment of regional SR agreements between cancer centers as well as designation of national centers of SR excellence that provide access to SR advanced technologies and services for members of multiple cancer centers. Additionally, respondents highlighted the ongoing challenge of defining and capturing SR return on investment and impact metrics (e.g., publications facilitated by SRs) in a standardized manner. This underscores both the operational burden of impact reporting and the need for enhanced NCI guidance related to P30 CCSG guidelines for SRs and SRM.

Notably, the results of this survey provide a first-of-its-kind benchmark of SR average percent cost recovery across cancer centers, offering immediately actionable context for SR leaders and institutional executives. For example, the average center-level SR percent cost recovery across cancer centers identified in this survey has the potential to help SR leadership at individual cancer centers calibrate expectations as they manage trade-offs between raising rates for higher cost recovery and ensuring cost-effective, ready accessibility to SR services for the members of their centers. This survey also provides a first-of-its-kind benchmark on SR advisory committees, including individual SR and center-wide SR mandates, membership composition, and meeting cadence. The survey results indicate the need to promote SRM best practices in areas like data management and priority access policies. Importantly, the results of this survey provide benchmarking metrics and information on common practices related to how SR-related decisions are made, including how key data is collected and what metrics are used to determine SR impact on cancer research.

Given the moderate sample size and incomplete response data, the results of this survey are not definitive and merit cautious interpretation and follow-up studies. Beyond describing standard practice, we interpret these patterns as hypothesis-generating to provide data and inform potential best practices for the community. As next steps, we will address key gaps in a follow-up survey which will include, for example, collecting percent cost recovery estimates by SR type (e.g., biostatistics and bioinformatics, genomics, flow cytometry, imaging, etc.) and contextualizing SR cost recovery by local cost of living and SR developmental stage.

The results of this survey should be useful for SRM leaders that oversee groups of cores as well as for leaders of individual SRs at cancer centers. The results are also applicable to optimizing core facility management at other types of institutions. Moreover, the results of this survey will be used by the NACCSR to provide recommendations to the NCI regarding the core metrics that are required in the individual SR sections and the centralized SRM sections of NCI P30 CCSG award applications.

### Conflict of Interest

The authors report no conflicts of interest.

### Statement of Research Involving Human or Animal Subjects

This study did not include research involving human or animal subjects
